# Hemophagocytic lymphohistiocytosis presenting in a pediatric patient with near total colonic and small bowel aganglionosis: a case report

**DOI:** 10.1186/s13256-017-1390-4

**Published:** 2017-08-31

**Authors:** Brittany Badal, Michael J. Wilsey, Sara Karjoo

**Affiliations:** Johns Hopkins All Children’s Hospital, 501 6th Ave S, St. Petersburg, FL 33701 USA

**Keywords:** Total colonic aganglionosis, Hemophagocytic lymphohistiocytosis

## Abstract

**Background:**

Total colonic and small bowel aganglionosis is a rare condition typically requiring intestinal transplant for long-term survival. There have not been any previously reported cases of near total intestinal aganglionosis complicated by concerns for hemophagocytic lymphohistiocytosis and need for both multivisceral organ transplant and hematopoietic stem cell transplant.

**Case presentation:**

Our patient is a 35-month-old Egyptian boy who presented with bilious emesis and failure to pass meconium shortly after birth. After evaluation, he was found to have near total colonic and small bowel aganglionosis up to the ligament of Treitz. When he was transferred to our tertiary facility, he was already diagnosed as having aganglionosis of total colon and partial small bowel whose case is complicated by the concern for hemophagocytic lymphohistiocytosis. He was not able to absorb any substantial nutrition enterally and was stabilized on long-term total parenteral nutrition which resulted in total parenteral nutrition-induced liver injury. While awaiting evaluation for liver and bowel transplant, he developed concerning symptoms consistent with hemophagocytic lymphohistiocytosis. He presents a complex challenge creating difficulty with management of whether to proceed with bowel transplant as a result of near-total intestinal aganglionosis or hematopoietic stem cell transplant for treatment of hemophagocytic lymphohistiocytosis. In this case, the transplant team proceeded with visceral transplant first, however he did not survive.

**Conclusions:**

This presentation of aganglionosis of total colon and partial small bowel complicated by the concern for hemophagocytic lymphohistiocytosis is unique to medical literature. For many physicians involved it is hard to determine how best to proceed with next steps in care.

## Background

Total intestinal aganglionosis is rare and severe, reported to occur in approximately 1% of all patients with Hirschsprung’s disease [1]. Similar to Hirschsprung’s disease, patients affected classically demonstrate inability to tolerate feeds, failure to pass meconium stool, bilious vomiting, and abdominal distention. Patients with near-total or total intestinal aganglionosis demonstrate a lack of ganglionic fibers extending throughout the submucosa of the colon [2]. In recent decades the use of long-term total parenteral nutrition (TPN) and increasing intestinal transplantation has improved survival opportunities for these patients. Total colonic and small bowel aganglionosis (TCSA) is a relatively uncommon variant, only occurring in 8 to 12% of patients with Hirschsprung’s disease [3]. It has also been linked to genetic mutations in the *RET* (rearranged during transfection) proto-oncogene, *EDNRB-EDN-3* (endothelin-3 gene), and the *SOX-10* (SRY-like homeobox 10) gene [4, 5]. Patients with total colonic aganglionosis with small bowel involvement have been noted to have increased mortality rates [2]. Here we present a unique case of pseudo-obstruction due to total aganglionosis of the colon and partial small bowel.

Our patient’s case has also been complicated by the diagnosis of acquired hemophagocytic lymphohistiocytosis (HLH) while undergoing workup for bowel and liver transplant. HLH is the abnormal overactivation of the immune system, typically related to macrophages and natural killer (NK) cells, leading to extensive inflammation and tissue damage. During the bowel transplantation evaluation for our patient it was noted that his liver enzymes were elevated disproportionate to what would be expected with only total TPN cholestasis. He acutely decompensated which prompted evaluation and recognition of HLH.

This combined condition of aganglionosis of the colon and small bowel as well as acquired HLH has never been reported in the literature. This has significantly affected transplant possibilities and it presents a management difficulty on the timing of either initiating bone marrow transplant versus multivisceral transplant.

## Case presentation

Our patient is a 35-month-old Egyptian boy who has a complex past medical history of total colonic and partial small bowel aganglionosis and acquired HLH. His birth history is significant for a twin gestation via *in vitro* fertilization to consanguineous parents. He lives primarily with his mother, father, and twin sister. There is no other family history of congenital abnormalities or of immune, hematologic, or gastrointestinal abnormalities in the family. The twin sister is healthy and developmentally normal.

On his first day of life, he was noted to have bilious emesis and failure to pass meconium stool. A rectal submucosal biopsy demonstrated a lack of ganglionic fibers raising concern for Hirschsprung’s disease. He subsequently underwent exploratory laparotomy with placement of an ileostomy on day of life 7. During this procedure he had mucosal biopsy samples taken from his rectum, sigmoid colon, cecum, and distal ileum. Each of which demonstrated total aganglionosis of the colon extending up to 6 cm above the ligament of Treitz. Due to inability to tolerate feeds, he was started on TPN. He was noted to appear icteric by 2 months of life and have an elevated bilirubin of 4.1 mg/dL as well as elevated transaminases including aspartate transaminase (AST) to 367 IU/L and alanine transaminase (ALT) to 387 IU/L on day of life 79. A liver biopsy performed at this time demonstrated ballooning hepatocytes, significant cholestasis, fibrosis, and bile duct proliferation consistent with TPN-induced liver injury.

After stabilization, he was transferred to a regional transplant center where he underwent evaluation for bowel transplant. As part of transplantation workup his initial hepatitis panel was found to be negative as was TORCH: toxoplasmosis, other (syphilis, varicella-zoster, parvovirus B19), rubella, cytomegalovirus (CMV), and herpes. He was noted during this time to have a chronic microcytic anemia despite multiple transfusions. Due to this chronic anemia, he subsequently underwent peripheral blood studies and bone marrow aspiration at 3 months of age. A bone marrow aspiration biopsy demonstrated myeloid hyperplasia. His microcytic anemia was proposed to be from chronic gastrointestinal blood loss and frequent venesection due to hypersplenism. He was stabilized and discharged home with continued follow-up while awaiting possible transplant.

There was also a new concern for disseminated primary CMV infection due to rising CMV titers after previous negative testing. He underwent repeat esophagogastroduodenoscopy, colonoscopy, and liver biopsy which demonstrated chronic gastritis and chronic liver injury consistent with TPN-dependence. His respiratory status continued to worsen; a bronchoalveolar lavage to investigate an infectious etiology was performed. He was found to have evidence of CMV, respiratory syncytial virus (RSV), Epstein–Barr virus (EBV), and *Pneumocystis* pneumonia infections. Due to concern for multivariate infections, including *Pneumocystis* pneumonia he underwent further immunodeficiency workup.

Despite treatment, he had persistent fevers and had little signs of clinical improvement. Due to this combination of prolonged fever, splenomegaly, cytopenias, hyperferritinemia, and hypertriglyceridemia, clinical suspicion for HLH was raised [6]. His laboratory studies demonstrated continued anemia and thrombocytopenia. Ferritin levels had steadily increased up to 4218 ng/mL. At this time his transaminases remained elevated with triglycerides increasing to 965 mg/dl. Due to clinical suspicion of HLH, a repeat bone marrow biopsy was performed demonstrating hemophagocytic histiocytes. He was found to have elevated plasma concentrations of soluble CD25 (soluble interleukin-2 receptor; sIL-2R) to 10,652 U/mL, a more specific marker for HLH. Due to history of consanguineous parents and concern for HLH, further genetic studies were performed to investigate a possible genetic etiology. These included *SAP*, *Perf1*, and *XIAP* which were found to have normal expression, lowering the likelihood of a familial known genetically linked HLH. It is proposed that his HLH was probably acquired secondary to EBV, CMV, or *Pneumocystis* infections.

Further genetic testing did demonstrate 360 Mb of homozygosity on microarray, although it did not contain any known genes for familial HLH. Due to high risk of mortality associated with HLH, he was started on etoposide and high-dose Decadron (dexamethasone) based on protocol HLH-2004. During this hospitalization, he was thought to be unresponsive to the HLH-2004 protocol, although the assessment of his response during this period is unclear. He remained unresponsive to initial therapies and was switched 2 months later to second-line treatment with basiliximab, with improvement in ferritin levels to 2750 ng/mL and interleukin-2 (IL-2) receptor inhibition with levels decreased to 197 U/mL. He has been discussed at multiple academic transplant centers for hematopoietic stem cell transplantation as well as for multivisceral solid organ transplantation. Due to his comorbidities, it was felt that he was not a transplant candidate at this time. The first transplant center discharged him home with supportive care.

At 29 months of age, he presented for acute care to our facility with difficulty ambulating and was subsequently found to have a right femur fracture. After surgical intervention for closed reduction of fracture, he developed severe respiratory distress and fever and was subsequently treated and evaluated for concerns of central line infection. During a prolonged hospitalization, he was noted to have progressive liver disease with worsening transaminases. His physical examination was notable for jaundice, bilateral scleral icterus, and significant hepatosplenomegaly. He was not mobile and would lay supine 24 hours a day. He had intermittent tachypnea due to the size of his distended abdomen. He also had a G-tube in place that required 24 hours of venting as he did not tolerate closure of G-tube. He also had a Broviac central line and required continuous TPN for nutritional support.

At presentation to our center, the hematology-oncology service was consulted and felt that although he had met clinical criteria for HLH he had not undergone further testing for specific HLH mutation or altered NK cell function. Further testing of his NK cytotoxicity function was obtained demonstrating overall normal function of NK cells, CD8, and CD56. Genetic testing for SAP expression, antigen stimulation, mitogen stimulation, and XIAP was also within normal limits. CD107A mobilization and expression was found to be decreased, which may indicate abnormal degranulation of NK cells. It has been concluded that the criteria he meets for HLH including anemia, hepatosplenomegaly, thrombocytopenia, hyperferritinemia, and liver failure are probably secondary to complications of near-total intestinal hypoganglionosis rather than a primary HLH as his overall NK cell function is within normal limits.

Further complicating this patient’s care, he has a prolonged history of recurrent bacteremia and urinary tract infections. He has regularly undergone infectious workup and antibiotic therapy on an almost monthly basis for much of his life. He has had long-term microcytic chronic anemia secondary to portal hypertension-related bleeding from his ileostomy site and has required irradiated red blood cell transfusions on a weekly basis. These problems coupled with his underlying near-total intestinal hypoganglionosis, pseudo-obstruction, chronic TPN-induced liver injury, and HLH create a difficult clinical conundrum.

He underwent evaluation by a second transplant institution for multi-organ transplant involving bowel, liver, and hematopoietic stem cell transplant (HSCT). After a multidisciplinary review of the bone marrow and organ transplant programs, a decision was made to proceed with visceral organ transplant first. However, he did not survive and had brain hemorrhage while on extracorporeal membrane oxygenation (ECMO).

## Discussion

Our patient presents a complex challenge creating difficulty with management: whether to proceed with bowel and liver transplant as a result of near-total intestinal aganglionosis or HSCT for treatment of acquired HLH. This poses a medical conundrum of whether to proceed further with transplantation and, if so, which transplant to attempt first, and/or whether it would be safe for such a complex patient to receive either transplantation. There is no reported case in the literature of a patient with near-total aganglionosis of the bowel and HLH presenting a transplant conflict.

Previously, the estimated incidence of HLH at pediatric tertiary care centers was 1 in 3000 in-patient admissions [6]. The estimated cross-sectional prevalence of HLH in Texas, USA, in 2010 was 1.07 cases per 100,000 persons less than 18 years of age [7].

Suspected HLH in our patient was stabilized with basiliximab, although a bone marrow transplant was pursued as a possible long-term treatment option. HSCT is typically indicated for patients with reactivation of disease or known genetic defects for improved long-term survival [8]. It has also been noted that transplantation outcomes are improved if a patient is in remission at time of transplant [8]. HSCT is complicated by the fact that both recipient and donor marrow have immunocompetent cells and the potential to reject each other, resulting in graft rejection or graft-versus-host disease (GVHD) [9]. Following HSCT transplant, infection is the leading cause of death early post-HSCT. Patients also require immunosuppressive therapy pretreatment with cytotoxic and myeloablative agents to prevent graft rejection [9], which may place our patient at even greater risk due to his history of recurrent infection and concern for possible immunodeficiency.

While some patients are successfully managed with surgical procedures that lead to an osteotomy and TPN, our patient has had chronic issues related to his osteotomy site and has TPN-induced liver injury related to his short gut syndrome with total aganglionosis of the colon extending up to 6 cm above the ligament of Treitz. Therefore, intestinal multivisceral transplant is required in such a patient for long-term survival, although this is not without risk. Most recent literature suggests improving success rates likely due to advances in technique and immune rejection treatment with an estimated 1-year patient survival rate at 80% [9] and 3-year patient survival rate improving at 65% [10]. Unfortunately, long-term survival has not improved with 5-year to 10-year survival remaining less than 50% [10]. Recipients of small bowel transplants are theoretically at high risk of rejection due to increased amount of lymphoid-containing tissue in the graft [11]. Mazariegos *et al*. demonstrated a series in which the rate of GVHD after intestine transplantation was 5.6%, which is notably more than the 1% GVHD rate following solid organ transplantation [11].

Rejection of the transplanted organ is of the utmost concern; intestinal transplant is fraught by increased risk of acute, late-onset, and chronic rejection. This is likely due to the intestine’s inherent heavy bacterial state and well-controlled inflammatory state. Also the intestine itself is a lymphoid organ and may play a unique role in the interaction between recipient antigen-presenting cells and donor graft tissue [12]. This raises concern for rejection leading to mucosal sloughing and bacteremia and death [10]. Patients who have an intestinal transplant are on varying different immunosuppressive therapies based on the transplant center, although typically they receive thymoglobulin or rabbit antithymocyte globulin (rATG) [12].

Unique to this patient is the need for both HSCT and multivisceral intestinal transplantation. In the literature there are instances of patients with both HSCT and solid organ transplant, although none that involve intestinal transplant. Schechter *et al*. reviewed pediatric transplant data spanning 2000 to 2009 and found that there were limited data on patients receiving HSCT and solid organ transplant [13]. Many of these patients received a secondary transplant due to either severe aplastic anemia following liver transplantation or post-transplant lymphoproliferative disease following solid organ transplant. Lazarus and Chiang examined patients that had a bone marrow transplant later followed by a solid organ, such as kidney, from the same donor and found that minimal immunosuppression was given and if rejection occurred it typically responded well [14]. It is important to note that a kidney transplant differs from liver transplants in that the liver graft may contain immunocompetent cells that can later cause GVHD in patients with a previous bone marrow transplant [14]. It was also observed that in this rare dual transplant patient population of six documented cases, there was no treatment-related mortality despite use of prolonged immunosuppressive therapy. Therefore, based on the current literature regarding bone marrow and solid organ transplant, initiating the liver and bowel transplant first before the HSCT may be more beneficial. More cases and studies need to be reviewed before physicians know the best approach to such complex patients.

## Conclusions

This case is unique to the literature due to presentation of aganglionosis of total colon and partial small bowel complicated by the concern for HLH. This case raises an interesting conundrum in determining an appropriate care plan given his comorbidities and history of medical complication. This case also highlights the importance of team-centered care and communication between all health providers in multiple centers as well as the family. At the time, since he had a multitude of comorbidities, the likelihood of success with such extensive transplantation was difficult to weigh against the risks of such procedures. For many physicians involved it is hard to determine how best to proceed with next steps in care. In our patient, organ transplant was considered first, however he did not survive. Further cases need to be evaluated and reported to help with management with future cases.

## Abbreviations

ALT: Alanine transaminase
AST: Aspartate transaminase
CMV: Cytomegalovirus
EBV: Epstein–Barr virus
ECMO: Extracorporeal membrane oxygenation
*EDNRB-EDN-3*: Endothelin-3 gene
GVHD: Graft-versus-host disease
HLH: Hemophagocytic lymphohistiocytosis
HSCT: Hematopoietic stem cell transplant
IL-2: Interleukin-2 receptor
NK: Natural killer
rATG: Rabbit antithymocyte globulin
*RET*: Rearranged during transfection
RSV: Respiratory syncytial virus
sIL-2R: Soluble interleukin-2 receptor
*SOX-10*: SRY-like homeobox 10
TCSA: Total colonic and small bowel aganglionosis
TORCH: Toxoplasmosis, other (syphilis, varicella-zoster, parvovirus B19), rubella, cytomegalovirus, and herpes
TPN: Total parenteral nutrition
